# Optimization of Topical Therapy for *Leishmania major* Localized Cutaneous Leishmaniasis Using a Reliable C57BL/6 Model

**DOI:** 10.1371/journal.pntd.0000034

**Published:** 2007-11-28

**Authors:** Hervé Lecoeur, Pierre Buffet, Gloria Morizot, Sophie Goyard, Ghislaine Guigon, Geneviève Milon, Thierry Lang

**Affiliations:** 1 Unité d'Immunophysiologie et Parasitisme Intracellulaire, Institut Pasteur, Paris, France; 2 Unité de Recherche Clinique, Institut Pasteur, Paris, France; 3 Plate-forme Santé Publique, Institut Pasteur, Paris, France; Hebrew University, Israel

## Abstract

**Background:**

Because topical therapy is easy and usually painless, it is an attractive first-line option for the treatment of localized cutaneous leishmaniasis (LCL). Promising ointments are in the final stages of development. One main objective was to help optimize the treatment modalities of human LCL with WR279396, a topical formulation of aminoglycosides that was recently proven to be efficient and safe for use in humans.

**Methodology/Principal Findings:**

C57BL/6 mice were inoculated in the ear with luciferase transgenic *L. major* and then treated with WR279396. The treatment period spanned lesion onset, and the evolution of clinical signs and bioluminescent parasite loads could be followed for several months without killing the mice. As judged by clinical healing and a 1.5-3 log parasite load decrease in less than 2 weeks, the 94% efficacy of 10 daily applications of WR279396 in mice was very similar to what had been previously observed in clinical trials. When WR279396 was applied with an occlusive dressing, parasitological and clinical efficacy was significantly increased and no rebound of parasite load was observed. In addition, 5 applications under occlusion were more efficient when done every other day for 10 days than daily for 5 days, showing that length of therapy is a more important determinant of treatment efficacy than the total dose topically applied.

**Conclusions/Significance:**

Occlusion has a significant adjuvant effect on aminoglycoside ointment therapy of experimental cutaneaous leishmaniasis (CL), a concept that might apply to other antileishmanial or antimicrobial ointments. Generated in a laboratory mouse-based model that closely mimics the course of LCL in humans, our results support a schedule based on discontinuous applications for a few weeks rather than several daily applications for a few days.

## Author Summary

When initiating the cutaneous disease named cutaneous leishmaniasis (CL), *Leishmania* parasites develop within the parasitophorous vacuoles of phagocytes residing in and/or recruited to the dermis, a process leading to more or less chronic dermis and epidermis-damaging inflammatory processes. Topical treatment of CL could be a mainstay in its management. Any improvements of topicals, such as new vehicles and shorter optimal contact regimes, could facilitate their use as an ambulatory treatment. Recently, WR279396, a third-generation aminoglycoside ointment, was designed with the aim to provide stability and optimal bioavailability for the molecules expected to target intracellular *Leishmania.* Two endpoints were expected to be reached: i) accelerated clearance of the maximal number of parasites, and ii) accelerated and stable repair processes without scars. A mouse model of CL was designed: it relies on the intradermal inoculation of luciferase-expressing *Leishmania*, allowing for *in vivo* bioluminescence imaging of the parasite load fluctuation, which can then be quantified simultaneously with the onset and resolution of clinical signs. These quantitative readout assays, deployed in real time, provide robust methods to rapidly assess efficacy of drugs/compounds i) to screen treatment modalities and ii) allow standardized comparison of different therapeutic agents.

## Introduction

Of the 350 million people exposed to the risk of *Leishmania* parasite inoculation and further development, 2 million each year experience the discomfort and potential complications of cutaneous leishmaniasis (CL). Many active lesions are disfiguring, and remain so when healing as inesthetic scars that expose patients to social stigma, sometimes for life [1],[2]. The demand for improved CL therapy has been fueled for decades by the lack of an efficient, affordable, easy-to-apply drug/schedule, as well as by the risks associated with the use of parenteral antiparasitic drugs such as pentavalent antimonial drugs or pentamidine [3],[4]. Topical therapy of CL is a promising approach [5],[6]. The aminoglycoside paromomycin is the most well studied compound as a potential topical treatment for CL [7]. First and second generation paromomycin-based ointments were either reasonably efficient [8],[9] but too irritant (first generation paromomycin-Methyl benzo chloride, “Leshcutan”) [10],[11] or well-tolerated but not efficient enough when first tested in humans (second generation paromomycin-urea “WHO formulation”) [12],[13]. WR279396, a third-generation aminoglycoside ointment that contains 15% paromomycin formulated in a hydrophilic vehicle as well as a second aminoglycoside, 0.5% gentamicin, was designed to be effective but non-irritative. This new formulation was recently shown to be efficient and safe for the treatment of *L. major* localized cutaneous leishmaniasis (LCL) (Ben Salah, Buffet *et al*.,submitted and [14]). Although very encouraging, this result is only one step toward a simple and easily applicable therapy for this neglected disease. Various parameters such as frequency and duration of application or application in the presence or absence of an occlusive dressing-may markedly influence the efficacy or safety of topically applied formulations [12],[13],[15],[16]. For example, once-a-day applications of Leshcutan are associated with less frequent and less severe local reactions than a twice-a-day application schedule [17]. Though still suboptimal, a 28-day schedule of paromomycin-urea (WHO formulation) is significantly more efficient than a 14-day schedule [12]. These 2 examples show that optimizing application parameters through clinical trials, the most reliable approach, takes years. Also, for obvious ethical reasons, there is usually no untreated control group in clinical trials, making interpretation of the mechanisms of drug action more difficult.

In order to more rapidly and accurately identify important parameters that influence the efficacy of WR279396, we designed and used a mouse model of CL that mimics important features of the natural sand fly dependent-transmission of parasites to mammal. A relatively low (10^4^) inoculum of *L. major* metacyclic promastigotes was injected in the C57BL/6 ear dermis [18]. As in a majority of patients with *L. major* CL [19], the development of localized dermal lesions in C57Bl6 mice is followed by spontaneous healing over the course of weeks to months [18]. Because luciferase transgenic parasites were used in this model, the kinetics of parasite load could be established without killing the mice: indeed, a linear correlation between bioluminescence values and parasite loads assessed by the reference limiting dilution technique has been previously established [20].

## Materials and Methods

### Mice

Female C57BL/6 (5 week old) and Swiss *nu/nu* mice were purchased from Charles River (Saint Germain-sur-l'Arbresle, France) and were housed under institutional guidelines of the A3 Animal facility at Institut Pasteur (Paris, France).

### Generation of bioluminescent *Leishmania major*

A 1.66 kbp *firefly luciferase* coding region was cut from pGL3 basic (Promega, Madison WI) using NcoI/EagI and subsequently cloned into the *Leishmania* expression vector pF4X1.HYG (Jenabioscience, Jena, Germany) with a marker gene for selection with Hygromycin B (Cayla, Toulouse, France) which was previously cut with NcoI/NotI. In this construct, 18s rRNA flanked the *luciferas*e and *HYG* genes. Following linearization with SwaI, *luciferase* and *HYG* genes were integrated into the 18s rRNA locus of the nuclear DNA of *Leishmania*. Transfections were realized by electroporation with the following conditions: 25 µF, 1500 V, in 4 mm cuvette; 3.75 kV/cm [21]. Following electroporation, cells were incubated 24 h in media without drug and plated on semisolid media containing 100 µg/ml of hygromycin B [20].

### Parasite preparation, inoculation in the ear dermis and parasite-loaded ear feature monitoring

Transgenic luciferase *L. major* strain NIH173 (MHOM/IR/-/173) amastigotes were isolated from infected Swiss nude mice. Briefly, the promastigote developmental stage was grown at 26°C in M199 media supplemented with 10% FBS, 25 mM Hepes pH 6.9, 12 mM NaHCO3, 1 mM glutamine, 1×RPMI 1640 vitamin mix, 10 µM folic acid, 100 µM adenosine, 7.6 mM hemin, 50 U/ml of penicillin and 50 µg/ml of streptomycin [21]. Infective-stage metacyclic promastigotes were isolated from stationary phase cultures (6 day old) using density gradient centrifugation, as previously described [22].

C57BL/6 mice were anaesthetised by intraperitoneal administration of a mixture of Ketamine (120 mg/kg^−1^ Imalgene 1000, Merial, France) and Xylazine (4 mg kg^−1^; Rompun 2%, Bayer, Leverkusen, Germany). Ten thousand metacyclic promastigotes per 10 µl of Dulbecco's phosphate buffered saline (PBS) were injected in the right ear dermis. Images of ketamine-xylasine anaesthetised mice were captured each day bioluminescence analyses were performed. The clinical features of parasite-loaded ear were examined based upon three phases: 1) early, leucocyte infiltrate-free inflammatory, processes, 2) leucocyte infiltrates-positive inflammatory processes and 3) late repair processes could be distinguished. Only one name, “lesion”, was used to designate these different processes. The “lesion” size measurement (mm^2^) was approximated from the picture by fit within a rectangle.

### *In vivo* bioluminescence imaging of the luciferase-transgenic *Leishmania* population size fluctuations in the ear dermis

Luciferin (D-Luciferin potassium salt, Xenogen, California), the luciferase substrate, was intra-peritonealy inoculated into mice at a concentration of 150 mg/kg 25 minutes before bioluminescence analysis. Mice were anaesthetised in a 2.5% isoflurane atmosphere (Aerane, Baxter SA, Maurepas, France) for 5 minutes and maintained in the imaging chamber for analysis. Emitted photons were collected by 1 minute acquisition with a charge couple device (CCD) camera (IVIS Imaging System 100 Series) using the high resolution (small bining) mode. Analysis was performed after defining a region of interest (ROI) that delimited the surface of the entire ear. The same ROI was applied to every animal at every time point. Total photon emission from the ventral image of each mouse ear was quantified with Living Image software (Xenogen Corporation, Almeda, California), and results are expressed in number of photons/sec/ROI. The photon signal from the ear is presented as a pseudocolor image representing light intensity (red = most intense and blue = least intense) and superimposed on the grey scale reference image. Of note, the lower threshold bioluminescence value indicates a parasite load of ≥5000 parasites per ear, precluding any detection of persisting parasite population that oscillates between 500 and 1000 parasites.

### Distribution of mice within groups on the basis of median bioluminescence values

Forty to 60 animals per experiment were inoculated with transfected *L. major* and the total photon emission of each ear was quantified 11 days later. Mice were monitored and distributed in groups according to an equal median bioluminescence value (1×10^6^–5×10^6^ photons/sec/ROI) and standard deviation. Each experimental group contained 7 to 10 mice, each individually ear-tagged (the contralateral ear with respect to the inoculation site).

### Topical formulation

Topical formulations were prepared at the Walter Reed Army Institute of Research (Washington DC). WR279396 consists of paromomycin sulphate (15%) plus gentamicin (0.5%) in a vehicle as previously described [23].

### Topical ointment regimen design

From day eleven post-*L. major* inoculation, topical ointments were applied to parasite-loaded ears once every two days for 10 days or once everyday for 5 days. Each formulation was applied using a sterile tip directly onto the ears to form a thin layer. Control groups were treated with the vehicle used in the medication without any of the active ingredients, i.e., the paromomycin and gentamicin. The ointment was either left open without dressing or covered with an occlusive dressing. The occlusive dressing was an adhesive polyurethane membrane (Tegaderm; 3M Health Care, St Paul, USA) that keeps water but is permeable to both water vapour and oxygen. Then two independent leaflets of 3M Micropore Surgical Tape (3M Health Care) were directly applied to the Tegaderm. This tape permitted maintenance of Tegaderm and formulation in contact with the ear during the two days.

### Quantification of parasites

We estimated the number of parasites present in parasite-loaded ears as previously described [24]. Ears were cut off. The dorsal ear half was separated from the cartilage-containing ventral ear half, cut into small pieces and ground in HOSMEM-II culture medium using a glass tissue homogenizer. Tissue/organ homogenates were serially diluted in HOSMEM-II culture medium and then dispensed into 96-well plates containing semi-solid agar (Bacto-Agar, Difco, Detroit, MI) supplemented with 10% sterile rabbit blood collected on heparin. Plates were incubated for ten days and each well was then examined and classified as positive or negative according to whether or not viable promastigotes were present. Limiting dilution analysis was then applied to the data to estimate the number of viable parasites, expressed in limiting dilution assay units (LDAU) [25]. Statistical analysis of the results was based on the maximal likelihood method [26],[27].

### Statistics

Lesion size or log transformed parasite loads were analyzed with a two-way analysis of variance (ANOVA). The two factors examined were the treatment (untreated, vehicle, drug, drug with occlusion) and the period of observation (treatment, post-treatment and final) in the statistical environment R. The assumption of homoscedasticity and normality were tested with the Bartlett and Kolmogorov-Smirnov test, respectively. If the interaction term was significant, pair wise comparisons using t tests were realized for each combination of factors. A probability level of *p*<0.05 was accepted for the purpose of declaring statistically significant treatment effects.

## Results

### Design of a reliable mouse model of LCL

The first objective of this study was to design and validate standardized readout assays for assessing different drug regimens using C57BL/6 mice inoculated with *Leishmania major.* To carry out these experiments, 10^4^ luciferase-expressing *L. major* metacyclic promastigotes were inoculated intradermally into the mouse ear. Parasites produced a significant bioluminescent signal *in situ* allowing parasite load expansion and reduction to be monitored non-invasively. The development and outcome of parasite burden and parasite-loaded ear features were examined simultaneously over a period of 3 months. The relationship between bioluminescence and the clinical features of the ear were respectively assessed by quantifying the number of photons per second per ear and measuring the “lesion” area.

**Figure 1:**
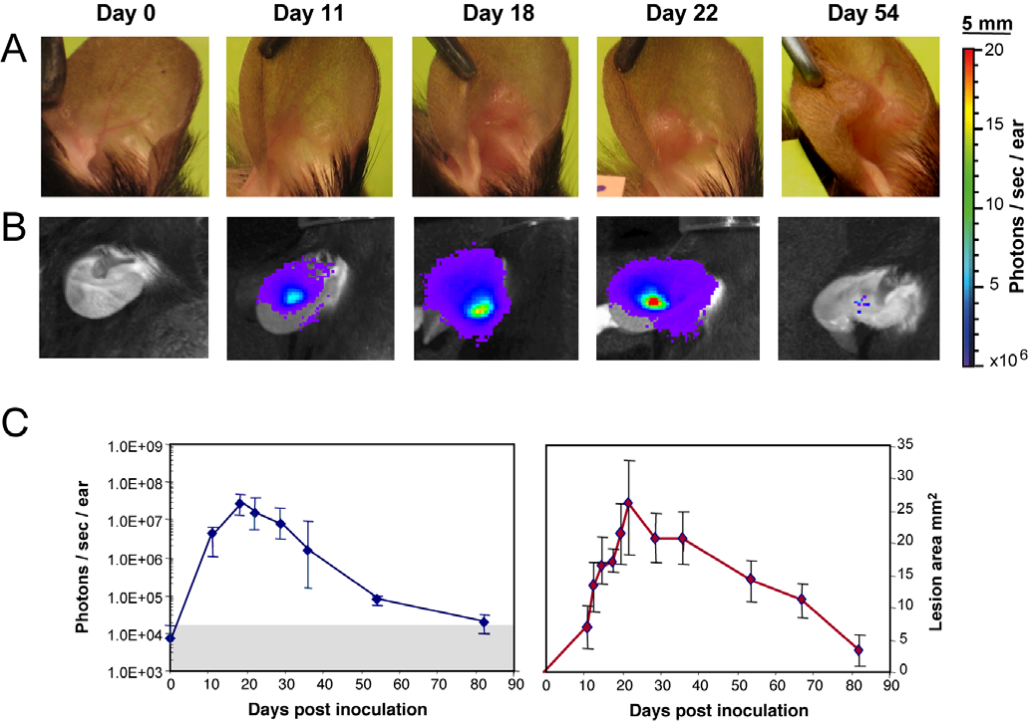
* In vivo* bioluminescence imaging of *Leishmania* in the C57BL/6 ear model. Simultaneous follow up of parasite load in the ear dermis and of lesion onset, features and cure in mice inoculated with luciferase-transgenic *Leishmania major*. 10^4^ luciferase-expressing NIH 173 metacyclic promastigotes were inoculated into the dermis of the right ear of C57BL/6 mice (day 0) and followed for more than 80 days. The bioluminescent signal is displayed as a pseudo-colour image representing light intensities over the body surface area. Red represents the most intense signal while blue corresponds to the weakest one. A, B: Individual follow up of a representative mouse left without any ointment application. Clinical features (A) were simultaneously monitored with parasite load fluctuations assessed by the bioluminescence of luciferase-expressing parasites (B). (C) Bioluminescence quantification of the parasite load (left panel; photons/sec/ear; grey area = background measurement) and “lesion” area (right panel; mm^2^) were followed for 7 mice left without any ointment and depicted as medians +/−sd. Note the detection of a bioluminescence signal before any significant clinically detectable features. Of note, the so called “lesion” area measured between day 11 and day 15 was still made up of inflammatory processes free of any leukocyte infiltrates.

Figures 1A and 1B illustrate the real-time bioluminescent images and clinical signs displayed by the *L. major*-inoculated ear from a representative C57BL/6 mouse (untreated group). The first post-inoculation phase (days 0–11) was characterized by a sharp increase of the bioluminescent signal at the inoculation site (from 7×10^3^ to 4.4×10^6 ^photons/sec/ear at day 11; Figure 1B, C). By day 7, mouse ears displayed no clinically detectable sign (Figure 1A). However, a leukocyte infiltrate-free tiny red spot (5 mm^2^) was observed at day 11 (Figure 1A, C). Thus, during the first stage of parasite development no significant correlation was found between the bioluminescence value at the inoculation site and the clinically detectable features. By day 22, the parasite load peaked (Figure 1B and 1C) with a median value of 1.5×10^7^ photons/sec/ear which was associated with the first *bona fide* cutaneous clinical signs (Figure 1A and 1C). The next phase of *L. major*-driven processes was characterized by a relatively sharp decrease in bioluminescence followed by healing of the ear lesion (Figure 1A, 1B and 1C). Following the complete and stable healing of this dermal lesion, no more bioluminescent signal was detected in the ear tissue (Figure 1B and 1C). We are aware that any persisting parasite load with a population size value ≤5000 per ear is not detectable using bioluminescence: thus, between days 80–96 post inoculation at the time of mouse sacrifice, mouse ears were recovered in the control and treated group. Using the LDA readout assay, these ears were monitored for the presence of persisting parasites. 40% of the ears were positive in all groups (≤500 parasites per ear) and these percentages were obtained from two independent experiments (data not shown).

In contrast, the persistent presence of a low number of parasites as measured by LDAU was noted in the inoculation site (3 positive mice out of 7) for up to 80 days post-inoculation.

These measurements helped us to define the onset of the first topical ointment application (WR279396 vehicle or WR279396 ointment with or without dressing). We decided to initiate treatment at day 11 post-inoculation for three reasons. First, at this time point, a high parasite load (bioluminescence values>1×10^6^ photons/sec/ear) was reproducibly measured. Secondly, these values were observed in the median part of acute-phase load, allowing for monitoring of either an increase in parasite load in the absence of any topical application or a decrease in treated groups. Thirdly, the last topical ointment application in the group of mice treated with WR279396 was coincident with the highest parasite load measured in the control group.

### Efficiency of WR279396 ointment under an occlusive dressing

**Figure 2:**
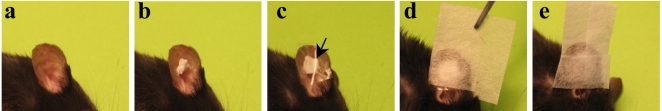
C57BL/6 mice given an application of WR279396 formulation under an occlusive dressing. 10^4^ luciferase-expressing *L. major* metacyclic promastigotes were inoculated into the dermis of the right ear of C57BL/6 mice (day 0). (a, b) From day eleven post-*L. major* inoculation, the topical ointment WR279396 was applied directly to parasite-loaded ears and (c) then covered with an adhesive polyurethane dressing (arrow). (d,e) Two independent leaflets of surgical tape were applied directly on the occlusive dressing. This surgical tape permitted maintenance of the dressing and kept the formulation in contact with the parasite-loaded site for two days.

**Figure 3:**
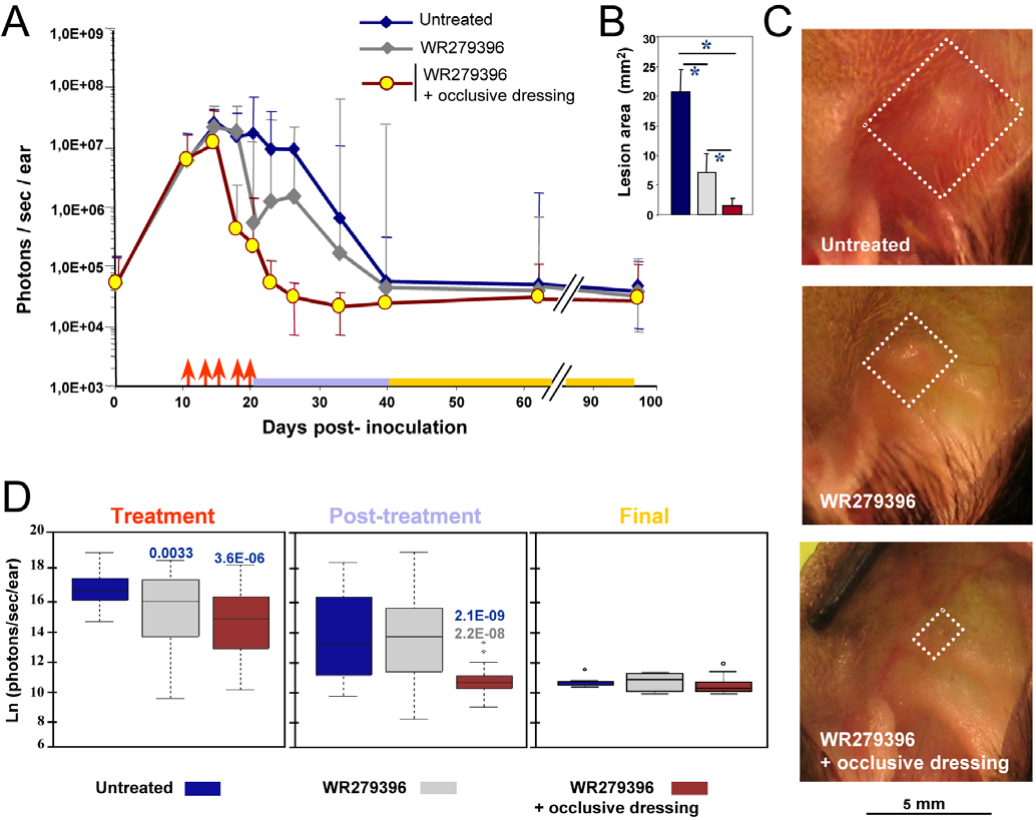
Earlier and sustained control of both parasite load and lesion healing post topical applications of WR 279396. 10^4^ luciferase-expressing *L. major* metacyclic promastigotes were inoculated into the dermis of the right ear of C57BL/6 mice (day 0) and mice were followed for 3 months. We compared parasite load and clinical feature outcome in mice left without any ointment (group 1, blue line) or receiving the WR279396 ointment (one application ( ↑ ) every two days, over ten days either without dressing (group 2, grey line) or with an occlusive dressing (group 3, brown line) at 11 day post-inoculation of promastigotes. Total photon emission from the *L. major*-loaded site (A, photons/sec/ear) is depicted as medians ±sd. Lesion area (B; mm^2^) and representative pictures of C57BL/6 *L. major* loaded-ears (C), at day 28 post-inoculation, are presented. Significant differences are indicated as follows: * for *P*<0.001. White squares delimit the “lesion” area of groups 1, 2 and 3 respectively. The three periods of observation (treatment, post-treatment and final) are represented by a colour code. (D) Comparison of bioluminescence (natural logarithm (ln) of photons/sec/ear) for each group of mice during the period of observation. The box plot for each group, assessed by a two-way ANOVA, graphs the percentile and median of parasite loads. The ends of the box define the 25th and 75th percentiles, with a line at the median and errors bars defining the 10th and 90th percentiles. The dots outside the ends of the whiskers are outliers. The *p*-values are displayed on the top of each box with the following colour code: -blue indicates that the group under study is compared to the blue group-grey indicates that the group under study is compared to the grey group.

By day 11 post-parasite inoculation, C57BL/6 mice were distributed in different groups on the basis of equal median bioluminescence values. WR279396 was applied topically to the *L. major*-inoculated ear every two days for 10 days. Occlusive dressing was performed by covering the *L. major*-loaded ears with adhesive polyurethane dressing (Tegaderm) and a surgical tape to maintain the formulation for 2 days (Figure 2). An evaluation of the effect of WR279396 with an occlusive dressing was monitored by measuring the bioluminescence and ear “lesion” area (Figures 3B and 3C). Three periods of observation have been defined i) the 10-day treatment period ii) the post-treatment period, which ends with the absence of any bioluminescence signals in the control group and iii) the late period. As controls, three groups were analysed. In the first group, ears were left untreated. In the second group, the paromomycin- and gentamicin-free vehicle was applied to *L. major*-inoculated ears that remained uncovered after application. In the third group, the WR279396 vehicle was applied and ears were immediately covered with an occlusive dressing. In all control groups, parasite load as well as lesion onset development and healing were simultaneously assessed. No statistical differences in parasite loads and lesion area were observed in any period between untreated and ointment vehicle-treated groups regardless of the period under study with respect to the measurement (not shown).

Monitoring of bioluminescence values showed that topical treatment with WR279396 (without a dressing) accelerated the decrease of both the parasite load (Figure 3A) and “lesion” area (Figure 3B and 3C). Two-way ANOVA analysis indicated a significant effect of treatment (*P-*value<9.2×10^−6^) and period (*p-*value<2.2×10^−6^) on parasite load for the whole experimental group, and there was a significant interaction between treatment and period effect (*p-*value<0.008). The parasite load (grey line; Figure 3A) decreased rapidly after the fourth application (day 18; Figure 3A). Median values of bioluminescence indicated that parasite loads in the group of mice left without dressing were significantly lower than the control group during the treatment period (Figure 3A and 3D; grey line vs blue line and box plot-: *p-*value = 0.0033).Of note, during the post-treatment period, a rebound pattern of parasite load was observed in mice treated with ointment without occlusive dressing (grey line; Figure 3A) no statistical difference between groups being noted (Figure 3D).

For mouse ears that were covered with WR279396 under an occlusive dressing, mean parasite loads (Figure 3A; brown lines) decreased earlier than those with WR279396 left without any dressing. One day post the last application, bioluminescence values reached threshold bioluminescence values (Figure 3A) in 80% of mice (8 out of 10). During the post-treatment period, statistical analyses indicated that parasite load in the group of mice with an occlusive dressing was significantly lower than in the group of mice treated without a dressing (Figure 3B, *p*-value = 2.2×10^−8^). The higher significant therapeutic effect of the drug in the presence of an occlusive dressing during this post-treatment period is illustrated in figures 3B, 3C (day 28: *p-*value = 0.00055). Furthermore, no rebound of parasite load was observed in this group. In conclusion, the decrease in parasite loads and the healing process occurred earlier in mice treated with WR279396 under an occlusive dressing. Among this group of mice, neither clinical relapse-as measured by leucocyte infiltrate-related “lesion” area-nor rebound of parasite load was detected.

**Figure 4:**
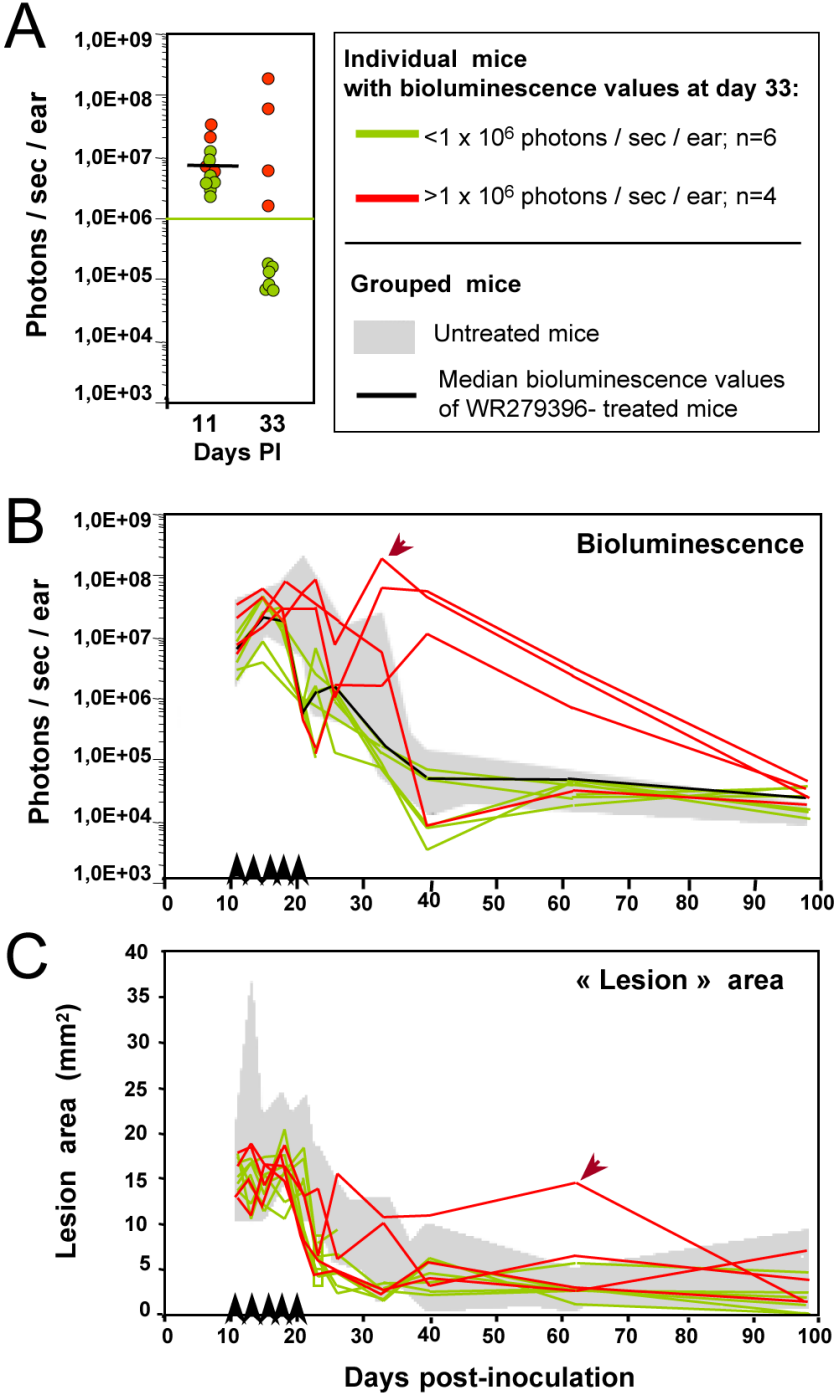
Individual follow up of bioluminescence (parasite load) and “lesion” area in mice treated with WR279396 without any dressing. 10^4^ luciferase-expressing *L. major* metacyclic promastigotes were inoculated into the dermis of the right ear of C57BL/6 mice (n = 10; day 0) treated with WR279396 (5 applications (↑) for 10 days) at day 11 post-inoculation. The parasite load (photons/sec/ear) in individual mice (A, B) and the area (mm2; C) of the lesion displayed by the same mice were followed for 3 months. (A, B, C) Green colour assesses the profile in mice that controlled their ear parasite load, i.e. exhibiting a bioluminescence value<1×10^6^ photons/sec/ear at day 33 (green points in panel A and green lines in panels B and C). In contrast, red colour corresponds to mouse ears that display a high bioluminescence value at day 33 (>1×10^6^ photons/sec/ear; red points in panel A and red lines in panels B and C). Note that lesion area values (C) did not assess any clinical failure except in 1 mouse (arrow). Values obtained for control mice are shown in the grey areas indicated in each graph.

Parasite rebound was observed in some mice given WR279396 without occlusive dressing. The individual follow-up of parasite-loaded mouse ears in real time indicated a clear dichotomy in the patterns of parasite load outcome (Figure 4; same experiment as Figure 3; n = 10). In the majority of treated mice, parasite load decreased faster than in control mice with a bioluminescence value lower than 1×10^6^ photons/sec/ear (Figure 4A; 6 out 10 mice depicted in green) at day 33. Furthermore, no rebound was detected in this group of mice (Figure 4B). In contrast, bioluminescence values for the four remaining mice were higher than 1×10^6^ photons/sec/ear at day 33 (Figure 4A and 4B; 4 mice depicted in red): post treatment, either the parasite load reduction pattern followed the same profile (1/4 mice; Figure 4B) as the one displayed by mice of the control group (grey area) or relapsed occurred at day 22 (3/4 mice; Figure 4B). The parasite load in this latter group of 3 mice remained higher than the parasite load of the control group from days 30 to 60. Interestingly, *bona fide* lesion area values did not assess any obvious clinical failure except in 1 mouse which harboured the highest parasite load (see arrow in Figure 4B) and displayed a somewhat slower healing process (Figure 4C; see arrow in Figure 4C). These data suggest that i) the rebound pattern of parasite load, which was observed in mice treated in the absence of occlusive dressing, had a clinical impact in a minority of mice and ii) the bioluminescence imaging data provided relevant information on parasite load fluctuations that were not provided by careful clinical monitoring.

### The optimal application regimen

**Figure 5:**
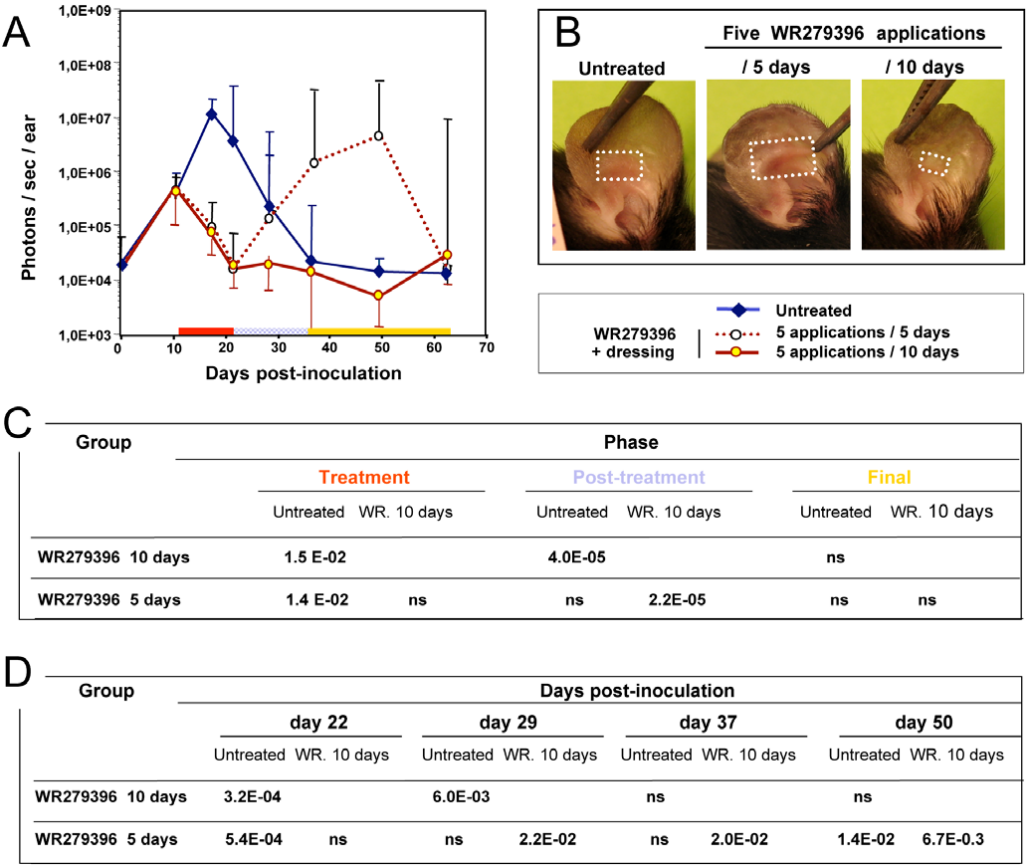
Determination of the most suitable regimen for WR279396 application under an occlusive dressing. Eleven days post-inoculation of 10^4^ luciferase-transfected *L. major* parasites, three groups of 7 mice were constituted: In the control group, mouse ears were left without any ointment (blue plain line), and in the other group WR279396 was applied on the mouse ear under an occlusive dressing with different application frequencies; either 5 applications/10 days (brown plain line) or 5 applications/5 days (brown dotted line). Bioluminescence evaluation of parasite load was performed over the course of 64 days post-inoculation (A). Note that the rebound of the parasite load in the every day-application group can be associated to a more severe lesion as illustrated by a representative mouse at day 36 post-inoculation (B/5 days). White squares delimit the “lesion” area. (C, D) Parasites loads were analysed with ANOVA whose two factors were the treatment (WR279396 10 days or 5 days) and the period of observation. Pair wise comparisons using t-tests were realized for each combination of factors.

We also monitored application regimens of WR279396 to determine which one might have a superior therapeutic index against the parasite. The experimental protocol shown in figure 5 was as previously described except for a different schedule of drug ointment application. At day 11, the topical ointment was applied on parasite-loaded ears either daily for 5 days or once every two days for 10 days. A control group (no medication) was used in parallel for determining comparability and efficacy of the different topical therapy regimens. As previously described, all parasite-loaded ears exposed to five applications for 10 days were healed by day 21 (Figure 5A) without relapse by day 64. All lesions (7/7) treated with WR279396 daily for 5 days had healed at day 21 (end of the topical therapy), but 71% (5/7) and 14% (1/7) of mice relapsed at day 50 and day 60, respectively. The clinical aspect of the lesions at day 36 post-inoculation (Figure 5B) confirmed the greater efficiency of the 5 every two days application over a 10 day schedule. By two-way ANOVA, it was shown that the difference between treatments depends on the observation periods considered (significant interaction term, *p-*value<0.05 ; Figure 5C). Pair wise comparisons using t-tests for each combination of factors are shown in figure 5D.

The integrative analysis of parasite load evolution in 4 experiments, involving mice receiving 5 applications for 10 days either with (n = 31) or without (n = 23) an occlusive dressing, shows that 74% of mice treated without a dressing controlled parasite loads (17/23) without relapse. Of the 6 remaining mice, 2 were unresponsive, as shown by parasite load values similar to untreated mice. The other mice initially controlled parasite loads and lesion size during the treatment period, but relapsed by day 30 as shown by parasite load values similar to or higher those measured from the ears of untreated mice. In contrast, 94% of mice treated with an occlusive dressing healed (29/31) by day 30. Only 6% (2/31) had detectable-though very low-parasite load (bioluminescence level<1×10^6^ photons/sec/ear) during follow-up. These results allow us to establish the greater parasitological efficacy of the schedule using an occlusive dressing, with a trend toward a prophylactic effect on relapse after a successful course of WR279396.

## Discussion

WR279396, a third-generation aminoglycoside-based ointment, was efficient on *L. major*-induced localized cutaneous lesions (LCL) in C57BL/6 mice. Five applications for 10 days under occlusion induced a 94% healing rate by day 30, without re-expansion of parasite loads. This high cure rate, as well as the general evolution profile in both treated and control mice, is strongly reminiscent of what has been observed in clinical trials (Ben Salah, Buffet *et al*.-submitted and [14]), providing a strong validation of this new model for drug-testing purposes.

The adjuvant use of an occlusive dressing significantly enhanced control of parasite loads. Several non-mutually exclusive mechanisms may account for these effects. First, the dressing prevented removal of the ointment from the lesion by protecting the skin from scratching, rubbing and scraping. These latter observations have been made in patients treated without occlusion, an important proportion of the ointment being wiped off by clothes during the day, sheets during the night or even attracted to a “protective” gauze put on the top of ulcerated lesions. Second, occlusion on burns or wounds favours epidermal regeneration (ie, ulceration closure). Finally, water retention by semi-permeable occlusive dressing (like the polyurethane film used here) results in hydration of the ointment application zone [28] and likely improves the penetration and diffusion of hydrophilic antiparasitic compounds into the dermis [15], where intracellular amastigotes multiply. The aminoglycosides paromomycin and gentamicin, the active ingredients in the WR279396 ointment, are OH-rich hydrophilic compounds. Whether the dominant mechanism of the adjuvant effect is merely mechanical (enough ointment maintained on the lesion) or linked to dermal diffusion issues, the occlusive dressing enhanced the healing process induced by active ingredients, and prevented persisting parasite loads to re-expand. Now that this adjuvant effect is established, future studies should be set up for dissecting its fine mechanisms.

Apart from a mild difference in thickness, LCL lesions in mice resemble human lesions both clinically and histologically. As opposed to systemic drug testing, topical drug testing in mice will be relevant since potential pharmacokinetic differences between mouse and human skin are expected to be minor and easily tractable to further analyses. These observations, along with the careful validation of the model, support the assumption that our results are likely to apply to human therapy. To our knowledge, occlusion has never been fully validated as an adjuvant for the topical therapy of human cutaneous lesions driven by invasive microorganisms, but several case reports have proposed this approach both in CL [29],[30] and non-infectious dermatologic conditions [15]. We provide here a strong validation of a concept that might apply to other antileishmanial or antimicrobial ointments. Ointments are usually painless, their application requires no sophisticated expensive device or local anaesthesia and they can be applied easily to both children and adults by a primary care health provider with minimal training. Excluding those *L. braziliensis.* foci where the incidence of mucosal extension can be high, ointment therapy of cutaneous lesions otherwise declared as neglected diseases should be favoured since the potential adjuvant effect of occlusion might help some ointment formulations to reach the required efficacy for development.

The duration of treatment is another important determinant of reaching a stable cure. A very short 5-day daily application schedule under occlusion led to a “rebound” pattern similar to that displayed in mice treated for 10 days without occlusion. In other words, too short of an application period may lead to parasite load rebound, this latter risk being partially controlled by an occlusive dressing. The ability to perform individual mouse follow-up revealed a dichotomic pattern of parasite load evolution (“sustained control” versus “unstable control with parasite rebound”), pervasive over many weeks post transient topical ointment application. Interestingly, these patterns were displayed over several weeks, i.e., well beyond the treatment application period. Parasite load level at the end of applications was not a good predictor of further evolution (Figure 4). So, not only parasite killing but also some modification of parasite environment determined the long-term outcome of tissue damage and repair processes. It is then very likely that, during the treatment application period, some integrated programs are triggered that will be the dominant determinant of evolution. Those results fit well with observations in human CL, such as the low prognosis value of parasitological tests at the end of therapy or the efficacy of therapeutic schedules stopped before lesion healing [4],[31]. Taken together, these experiments show that parasites must be exposed to the drug for>5 days to drive evolution toward long term sustained control of parasite loads and clinical healing. Duration of drug exposure was a stronger determinant of outcome than the total amount of drug used. Intracellular pharmacokinetics of aminoglycosides helps understand the mechanism leading to this observation. In eukaryotic cells exposed to aminoglycosides *in vitro*, a slow (2–4 days) lysosomal accumulation is observed, followed, when aminoglycosides are removed from the extracellular medium, by an even slower (2–5 days) release [32]. Interestingly, the lysosome is the only subcellular compartment in which aminoglycosides accumulate, an important feature of their antileishmanial efficacy [32],[33]. So, provided that appropriate concentrations of aminoglycosides are reached in the dermal intercellular space, relatively discontinuous applications would probably suffice to allow intracellular killing of replicating amastigotes and long term sustained control of parasite loads. Taken together, our observations will help select the most efficient ointment application schedules for implementation, in the context of the therapy of this neglected disease, by health care providers with little resources and heavy duties. Even relatively discontinuous applications for a few weeks should be preferred to many daily applications for a few days.

Our model offers relevant preclinical readout assays i) of the efficacy of a topical ointment delivered under occlusion or not ii) for establishing the proper regimen/schedule that allows sustained parasite load reduction and lesion healing during post-treatment period features. This luciferase-based imaging study might be useful for pre-clinical evaluation of novel formulations containing molecules that target parasite-loaded cells residing in the dermis as well as molecules that contribute to damaged skin-repair processes. Next challenges will be to screen molecules expected to act on the amastigote population that persist in the dermis or in distant sites [34] and to investigate the acquisition in real time of long-term protective immunity.
